# Comparing the mental health trajectories of four different types of keyworkers with non-keyworkers: 12-month follow-up observational study of 21 874 adults in England during the COVID-19 pandemic

**DOI:** 10.1192/bjp.2021.205

**Published:** 2022-01-19

**Authors:** Feifei Bu, Hei Wan Mak, Daisy Fancourt, Elise Paul

**Affiliations:** Department of Behavioural Science and Health, Institute of Epidemiology & Health Care, University College London. UK; Department of Behavioural Science and Health, Institute of Epidemiology & Health Care, University College London. UK; Department of Behavioural Science and Health, Institute of Epidemiology & Health Care, University College London. UK; Department of Behavioural Science and Health, Institute of Epidemiology & Health Care, University College London. UK

**Keywords:** COVID-19, pandemic, depression, anxiety, keyworker

## Abstract

**Background:**

There are concerns that keyworkers may be at a greater risk for psychological distress than non-keyworkers during the COVID-19 pandemic. However, little research has included keyworkers outside of the healthcare sector or has disaggregated keyworkers into different subgroups.

**Aims:**

To examine longitudinal changes in mental health over 12 months during the COVID-19 pandemic comparing four different groups of keyworkers with non-keyworkers.

**Method:**

Longitudinal data were from 21 874 adults living in England (21 March 2020 to 22 February 2021). Latent growth modelling was utilised to compare growth trajectories of depressive and anxiety symptoms in non-keyworkers and four types of keyworkers: (a) health and social care workers, (b) teachers and child care workers, (c) public service workers, and (d) essential services keyworkers (such as food chain or utility workers).

**Results:**

When accounting for both time-invariant and time-varying covariates, keyworkers in the essential services category had consistently higher levels of depressive and anxiety symptoms than non-keyworkers across the whole of the study period. There was little difference in the mental health trajectories between health/social care, teachers/child care and public service worker categories and non-keyworkers.

**Conclusions:**

Our findings suggest that the risk for poorer mental health during the COVID-19 pandemic varies within the broad category of keyworkers generally, and that those working in utility, food chain and transport roles are especially at risk. Future research should focus on identifying which aspects of working conditions may be contributing to occupational stress in these groups.

## Background

The COVID-19 pandemic has had substantial detrimental effects on public mental health.^1^ Certain subgroups such as keyworkers (for example individuals working in the healthcare and social support sectors, delivery workers, teachers) have been posited to be more adversely affected than the rest of the population.^2^ However, despite keyworkers comprising a significant proportion of the population (33% in the UK),^3^ and fulfilling a large variety of roles with differing levels of exposure to the public and thus to the virus itself,^4^ the majority of the research on keyworkers’ mental health has focused exclusively on healthcare workers^5, 6^ or has examined keyworkers broadly as a collective.^7-10^ Keyworkers in general have been found to be more likely than non-keyworkers to meet criteria for clinically significant mental distress^9^ and probable criteria for depression, anxiety and post-traumatic stress disorders than non-keyworkers.^11^ However, other studies conducted during the COVID-19 pandemic have not found keyworkers to report more depressive or anxiety symptoms than non-keyworkers.^7,8,12^ These equivocal findings may be because of the heterogeneity involved when grouping all keyworkers together. For example, even among the specific category of healthcare workers, those whose jobs require direct contact with patients who have COVID-19 display more symptoms of anxiety, depression, insomnia and traumatic stress than healthcare workers not working directly with patients who have COVID-19.^5,6^

These varied findings may also be because of the variation in the amount of stress experienced across other keyworker groups in different sectors. There may have been a disproportional impact on the mental health of keyworkers fulfilling roles other than in healthcare during the current pandemic for several reasons. First, like healthcare workers, they have also been required to leave their homes for work despite the associated risk of infection and mortality in themselves and in family and friends.^4,13^ They may have also been challenged by longer working hours and more intense working circumstances, sometimes with inadequate personal protective equipment (PPE),^14,15^ the latter of which has been found to be associated with depressive and anxiety symptoms among keyworkers during the current pandemic.^16^ Degree of exposure to the public and therefore to the virus may also correlate with increased distress in other keyworker roles as well. One US study conducted in the first few months of the pandemic found that grocery store workers who interacted directly with the public were more anxious and depressed than those who did not interface with customers.^17^ Some keyworkers may additionally have already been at greater risk for severe infection and mortality from COVID-19 because of their age, pre-existing health conditions, living in areas of high socioeconomic deprivation and belonging to an ethnic minority group.^4,13^

So far only two studies have disaggregated and compared the mental health of different keyworker groups other than in the healthcare sector. In the first few months of the pandemic in the UK, one study found that food workers were the only keyworker group of the eight groups studied that were more likely than the others (for example utility workers, transport workers, health and social care workers) to meet probable criteria for anxiety disorder.^11^ In the same study, all keyworker groups besides transport workers were more likely to meet criteria for probable post-traumatic stress disorder than non-keyworkers, and odds for meeting this criterion varied substantially, from 1.7 in health and social care workers to 3.4 in public service workers.^11^ A second study conducted in Australia reported keyworkers other than in the health-care sector to have higher levels of anxiety, depression, stress and poorer quality of life than healthcare workers and the rest of the population.^18^

### Aims

Together, these findings suggest that although keyworkers broadly may be more vulnerable to experiencing poorer mental health than the rest of the population during the COVID-19 pandemic,^9,11^ keyworkers in other roles may be more at risk than those in the healthcare sector.^11,18^ However, research on the longitudinal changes to mental health of the keyworkers as the COVID-19 pandemic develops is lacking. It is also unknown whether mental health trajectories vary among different keyworker groups. Therefore, the aim of this study was to compare the growth trajectories of anxiety and depressive symptoms of four categories of keyworkers with non-keyworkers over the first 12 months (March 2020 to February 2021) of the pandemic in the UK. The findings will inform our understanding of which specific keyworker groups may be most in need of psychosocial support during the current and in future pandemics. Further, given that the effects of the COVID-19 pandemic on mental health could be long lasting, it is crucial to identify the various mental health experiences among keyworkers that could help in designing policies and interventions to support those who may continue to be affected by the COVID-19 crisis in post-pandemic times.

## Method

### Study design and participants

This study analysed data from the University College London (UCL) COVID-19 Social Study; a large panel study of the psychological and social experiences of over 75 000 adults (aged ≥18 years) in the UK during the COVID-19 pandemic. The study commenced on 21 March 2020 and involves initially weekly and then monthly (4-weekly) online data collection from participants for the duration of the pandemic. The study did not use a random sample design and therefore the original sample is not representative of the UK population. However, it does contain a heterogeneous sample that was recruited using three primary approaches.

First, convenience sampling was used, including promoting the study through existing networks and mailing lists (including large databases of adults who had previously consented to be involved in health research across the UK), print and digital media coverage, and social media. Second, more targeted recruitment was undertaken focusing on (a) individuals from a low-income background, (b) individuals with no or few educational qualifications, and (c) individuals who were unemployed. Third, the study was promoted via partnerships with third-sector organisations to vulnerable groups, including adults with pre-existing mental health conditions, older adults, carers and people experiencing domestic violence or abuse.

The study was approved by the UCL Research Ethics Committee [12467/005] and all participants gave informed consent. A full protocol for the study is available online at www.COVIDSocialStudy.org.

The research questions in the UCL COVID-19 Social Study built on patient and public involvement as part of the UKRI MARCH Mental Health Research Network, which focuses on social, cultural and community engagement and mental health. This highlighted priority research questions and measures for this study. Patients and the public were additionally involved in the recruitment of participants to the study and are actively involved in plans for the dissemination of findings from the study

To examine trajectories of mental health in relation to specific containment measures, we focused solely on participants who lived in England (*n* = 58 624) because these measures varied considerably across countries in the UK. We were interested only in keyworkers who were employed, with non-keyworkers as the reference category. Thus, the unemployed or economically inactive were excluded from the analysis (*n* = 19 369). Further, we included only participants with at least three repeated measures between 21 March 2020 and 22 February 2021. These criteria provided us with data from 22 012 participants. Around 1% of these participants withheld data or preferred not to self-identify on demographic and health-related factors and were therefore excluded from our analysis. This provided us with a final analytic sample size of 21 874 participants who were followed up for a maximum of 12 months.

### Measures

#### Keyworker status

When participants first joined the study, they were asked if they were currently fulfilling any of the government’s nine identified keyworker roles. These were categorised into five groups:

(a) non-keyworker (reference category);
(b) health, social care or relevant related support worker;
(c) teacher or child care worker;
(d) public service worker (for example justice staff, religious staff, public service journalist or mortuary worker, local or national government worker); and
(e) essential services keyworker (for example food chain, utility, public safety or national security worker, worker involved in medicines or protective equipment production or distribution).

#### Outcome variables

Depressive symptoms were measured using the Patient Health Questionnaire (PHQ-9);^19^ a standardised instrument for screening for depression in primary care. Unlike the original PHQ-9, the current study enquired about symptoms ‘over the last week’ instead of ‘over the last two weeks’ as data were initially collected weekly. The questionnaire includes nine items with four-point responses ranging from ‘not at all’ to ‘nearly every day’. Higher overall scores indicate more depressive symptoms, ranging from 0 to 27.

Anxiety symptoms were measured using the Generalized Anxiety Disorder assessment (GAD-7);^20^ a well-validated tool used to screen for generalised anxiety disorder in clinical practice and research. These questions were also worded as ‘over the last week’ for the same reason as the depression items. The GAD-7 comprises seven items with four-point responses ranging from ‘not at all’ to ‘nearly every day’, with higher overall scores indicating more symptoms of anxiety, ranging from 0 to 21.

These data were collected weekly between 21 March and 21 August 2020, and then monthly (4-weekly) starting from 24 August 2020 onwards (see Supplementary Table 1 and 2 available at https://doi.org/10.1192/bjp.2021.205). Our analyses used months as the unit of time. Mean values of depressive and anxiety symptoms across 4 weeks were used when data were collected weekly.

#### Time-invariant covariates

A range of sociodemographic and health factors were considered as potential confounders. These included gender (women versus men), ethnicity (White versus ethnic minorities), age groups (age 18-29, 30-45, 46-59, ≥60) and education (up to General Certificate of Secondary Education (GCSE), Advanced Level qualifications (A-levels) or equivalent, and university degree or above).

We included two health-related factors: self-reported diagnosis of any long-term physical health condition (such as asthma or diabetes) or any disability (yes versus no) and self-reported diagnosis of any long-term mental health condition (such as depression, anxiety) (yes versus no). All of these were time-invariant covariates that were measured when participants first joined the study.

#### Time-varying covariates

First, we considered a time-varying covariate to indicate if participants had gone to work outside of the home (yes/no). To provide consistent measurement between the weeks of the study when participation was weekly versus monthly, we coded this variable as whether participants worked outside at any point during the prior 4-week period.

Second, in December 2020 the UK began its COVID-19 vaccination programme and health and social care workers were one of the priority groups to be vaccinated. Therefore, we considered another time-varying covariate indicating if participants had had their first dose of the vaccine (yes/no) from December 2020 onwards (months 10-12).

### Statistical analysis

Data were analysed using the latent growth modelling (LGM) approach. We used unspecified LGM that allows the shape of growth trajectories to be determined by data by using free time scores. In this model, two time scores were fixed at 0 and 1 for the purpose of model identification, whereas the rest were estimated freely, allowing for an empirically based non-linear shape for the outcome growth trajectory. In models for depressive and anxiety symptoms, keyworker status and other time-invariant covariates were allowed to predict the growth factors (intercept and slope; Model I). Then, we added the time-varying covariates allowing them to predict the outcomes directly (Model II). The full model specification is presented in Supplementary Figure 1.

Weights were applied throughout the analyses. The final analytical sample was weighted to the proportions of gender, age, ethnicity and education among adults in employment in England based on the Quarterly Labour Force Survey (March-May 2020).^21^ Main analyses were implemented in Mplus Version 8.

## Results

### Descriptive characteristics

In the unweighted analytic sample of 21 874 participants, women (78.8%) and people with a university degree or above (75.2%) were overrepresented, whereas younger adults (aged 18-29; 6.7%) and people from ethnic minority groups (5.4%) were underrepresented (Table 1). After weighting, the sample reflected population proportions, with 51.0% women, 39.4% participants with a university degree or above, 15.3% aged under 30 and 10.9% of participants belonging to an ethnic minority group.

As shown in Table 1, demographic and health characteristics differed across the keyworker groups (see Supplementary Table 3 for unweighted results). For example, although there were more women than men in the ‘teacher/child care’ category (74.2% *v*. 25.8%) and in the ‘health/social care’ category (69.8% *v*. 30.2%), the gender proportions in non-keyworkers and public service workers were similar. Only 15.0% of keyworkers in the essential services category had a degree or above, this percentage was 35.8% among public service keyworkers, 41.9% among non-keyworkers, 47.1% among health and social care workers and 51.6% among teachers or child care workers.

**Fig. 1:**
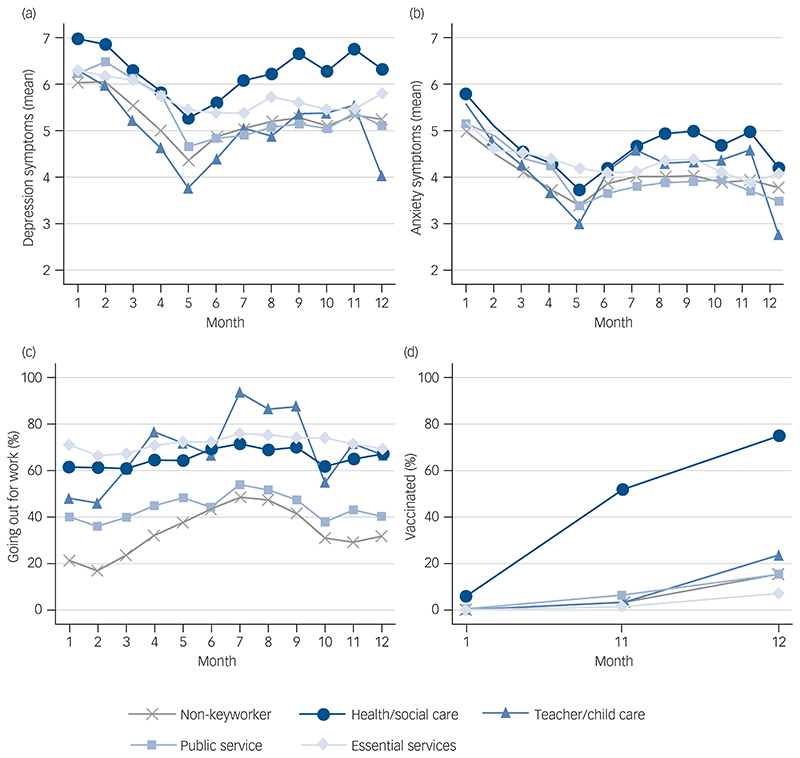
Descriptive statistics of the time-varying variables across time points by keyworker status. (a) Depressive symptoms; (b) anxiety symptoms; (c) going out for work; and (d) vaccinated. Depressive symptoms were measured with the Patient Health Questionnaire-9 and range from 0 to 27, and anxiety symptoms were measured with the Generalized Anxiety Disorder-7 and range from 0 to 21.

Figures 1(a) and 1(b) depict changes in the mean level of depressive and anxiety symptoms, respectively, and suggest some differences in the longitudinal changes in mental health by keyworker status (see Supplementary Table 4 for categorised measures). Fig. 1(c) shows the percentages of participants who went outside their homes for work in each keyworker category across different time points. Generally speaking, key-workers were more likely to have left home for work than non-keyworkers, especially teachers (when schools reopened in the autumn 2020) and keyworkers in the essential services category. The latter group seemed to have left home for work most consistently over the course of the study period. As expected, the percentage of those who were vaccinated from December 2020 onwards increased dramatically for health and social care workers (Fig. 1(d)). By contrast, keyworkers in the essential services category had the lowest level of vaccination across all groups including non-keyworkers, with the gaps appearing to widen over time.

### LGM

**Fig. 2:**
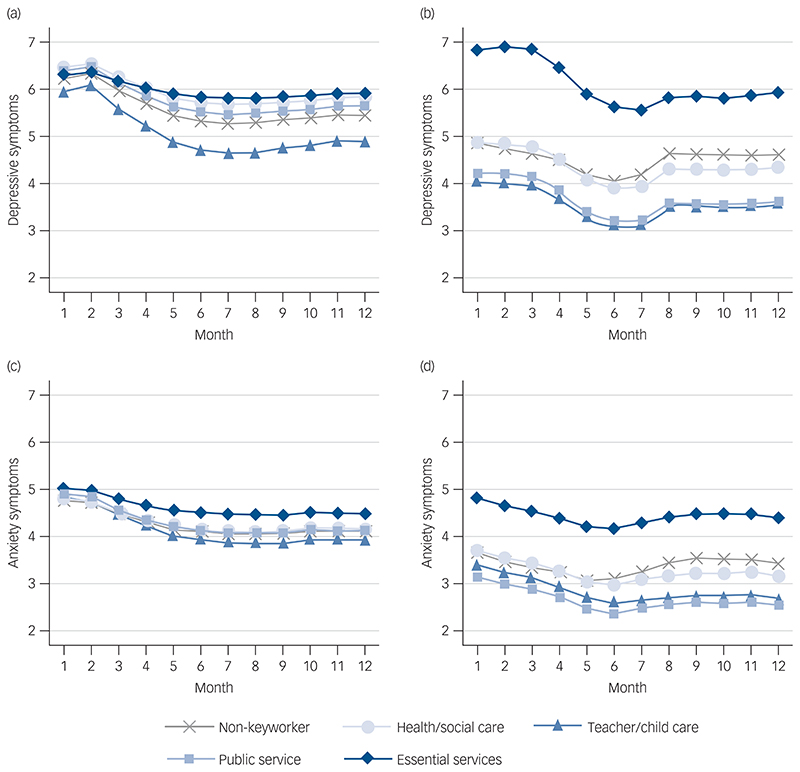
Predicted growth trajectories of depressive and anxiety symptoms by keyworker status from latent growth models. Model I controlled for time-invariant covariates: (a) depressive symptoms and (c) anxiety symptoms; Model II controlled for both time-invariant and varying covariates: (b) depressive symptoms and (d) anxiety symptoms. Depressive symptoms were measured with the Patient Health Questionnaire (PHQ-9) and range from 0 to 27, and anxiety symptoms were measured with the Generalized Anxiety Disorder-7 rand range from 0 to 21.

Overall, depressive and anxiety symptoms were worst at the start of the pandemic but then improved as restrictions from the first lockdown eased over the summer before worsening as COVID cases increased again in the autumn of 2020. There was little evidence that the intercept or slope of depressive symptom growth trajectories differed by keyworker status when only controlling for time-invariant sociodemographic and health factors (Fig. 2(a), see also Supplementary Table 5 and 6 in the Supplementary Materials for full results). However, after controlling for the time-varying factors (leaving the house for work and COVID-19 vaccination status), keyworkers in the essential services category had significantly higher depressive symptoms at the start of the observational period (intercept) compared with non-keyworkers (Fig. 2(b)). This was mainly because of the introduction of leaving home for work, which was associated with higher levels of depressive symptoms at the start (month one) but reduced depressive symptoms between months 4 and 7 (Supplementary Table 5).

There was no evidence that having had the COVID-19 vaccine was associated with changes in depressive symptoms. The difference in the intercept between teacher/child care workers and non-keyworkers was also statistically significant (*P* = 0.042, Supplementary Table 5). Specifically, teacher/child care workers had fewer depressive symptoms at the start of the study. There was little difference in slopes across keyworker status. Keyworkers in the essential services category had more, and teacher/child care workers had fewer depressive symptoms than non-keyworkers consistently across the 12-month observational period.

The growth trajectories for anxiety symptoms were similar between non-keyworkers and each of the keyworker categories when only including time-invariant covariates (Fig. 2(c)). However, after controlling for time-varying covariates, in particular leaving home for work, keyworkers in the essential services category had more anxiety symptoms compared with non-keyworkers and other keyworker groups across the entire study period. There was little difference in the slopes across the remaining keyworker groups. Similar to the results for depressive symptoms, essential services keyworkers had a consistently higher score of anxiety symptoms than non-keyworkers across the observation period.

## Discussion

### Main findings

Our results show that keyworkers in essential service sectors (for example food chain, utility, transport and public security or safety) had consistently higher depressive and anxiety symptoms over the entire 1-year study period. In contrast, teacher/child care workers had fewer depressive symptoms than non-keyworkers at the start and for the duration of the study. Despite the disproportionate research attention paid to the mental health of healthcare keyworkers during the pandemic, we did not find evidence that those working in the health/social care and public service sectors had levels of depressive and anxiety symptoms that were higher than non-keyworkers.

### Explanation for our findings

This is now the third study to have found worse mental health outcomes in keyworkers employed in sectors other than healthcare.^11,18^ In our study, keyworkers within essential services reported consistently higher levels of depressive and anxiety symptoms than non-keyworkers independent of potential confounding factors. There are a number of potential explanations for why this group may have been particularly badly affected. First, qualitative studies of such keyworkers have documented particular challenges such as adapting to duties under more stressful circumstances (such as increased workloads and fears of transmitting the virus to family members^15^). Second, individuals in essential services keyworker roles (such as transport, postal and retail workers) have received less recognition from the public or their employers for their efforts, which could have resulted in a feeling of inadequacy and further exacerbated their mental health.^15^ Third, essential services keyworkers are disproportionally more likely to have lower levels of educational attainment, to be in more routine occupational roles and to experience financial hardship than other categories of keyworkers and the general population. Studies focusing on the experiences of people in roles of lower socioeconomic position have consistently shown poor mental as well as physical health,^22^ and these findings could therefore reflect existing socioeconomic health inequalities within society.^23^ Finally, keyworkers in essential services roles have also been at particularly high risk for contracting COVID-19.^4,13^

It is notable that the distinctions between the mental health experiences of essential services keyworkers and other keyworker roles were exacerbated when taking into account having to work outside the home, suggesting that exposure to risk may have had an adverse effect on mental health. This theory is also supported by qualitative work highlighting the challenges faced by essential services keyworkers in often being unable to access PPE through their employers in comparison with other keyworker groups such as health/social care workers and teachers where although challenges with PPE remained, there was more attention given to safe working environments.^15^

Another key finding from this study was that health and social care workers did not show higher levels of anxiety or depressive symptoms than non-keyworkers, although their levels were descriptively higher than some other groups such as teachers/child care keyworkers and public service keyworkers. However, our findings should not necessarily be taken to imply that mental health has not been adversely affected among health and social care workers. First, this study looked just at symptoms of anxiety and depression and only measured from the start of the first lockdown in 2020. By this point, hospitals were already overwhelmed with patients who had COVID-19. As we lack data on mental health in this group prior to the pandemic, it is therefore unclear whether symptoms recorded here were higher than usual baselines. Second, this study looked at general symptoms of anxiety and depression, but other studies have suggested effects on wider aspects of mental health that these measures may not have captured such as post-traumatic stress.^11^ Finally, our category combined the experiences of health and social care workers without differentiating between front-line workers versus those working in other roles that might not have involved exposure to patients with COVID-19. As documented in other studies,^5,6^ the nature of health and social care roles within the pandemic has been shown to have differential effects on mental health. Therefore, future studies are encouraged that explore in more depth the types of health and social care roles that may have been most adversely affected.

It is also notable that keyworkers in the teacher/child care sector appeared to have fewer depressive symptoms than non-keyworkers at the beginning of the pandemic in March 2020 and over the course of follow-up, although the differences were slight. There are several possible explanations for this. First, the start of lockdown in 2020 in the UK involved the closure of schools. This reduced the workload for many in these professions as most schools were unable to deliver a full curriculum online and also reduced chances of exposure to the virus among this group. At the same time, teachers did not face the stress of furlough schemes or unemployment as their jobs were recognised as essential when society did reopen, which may contrast with non-keyworker sectors where a greater proportion of people were furloughed. Nonetheless, some other workers within this keyworker category such as nursery workers did experience furlough and redundancy. As the pandemic continued and schools reopened, teaching and child care then offered an opportunity to maintain face-to-face social interactions with a population that is considered less likely to be affected by the virus (children and adolescents),^24^ which could have helped improve their well-being during lockdowns and when the strictest physical distancing measures were in place.

Our study did not find any statistical differences between the mental health of public service workers and non-keyworkers. Many in the former group may enjoy greater job security and lower furlough rates, as well as the ability to work from home. Although many non-keyworkers may have also been able to work from home, they may have been faced with fears of job losses, financial concerns and stress because of not being able to leave home for work. Although the aim of this study was to report trajectories rather than prevalence, non-keyworkers evidenced anxiety symptoms that were higher than averages in other studies pre-pandemic using the same measure (2.7-3.2^25^). These findings point to the importance for monitoring and supporting mental health in the population as a whole in the current and in future pandemics.

### Strengths and limitations

This study has a number of strengths. It utilised a large sample with sufficient heterogeneity to include good stratification across all major sociodemographic groups. The analyses were weighted on the basis of population estimates of core demographics, with the weighted data showing good alignment with national statistics from the Labour Force Survey;^21^ a nationally representative study. As a result of the richness of the dataset, we were able to employ advanced statistical analyses to examine the trajectories of depressive and anxiety symptoms among keyworkers in various sectors since the first lockdown in the UK across different stages of the pandemic over 12 months. Despite these strengths, the limitations of our study raise important points for future research on mental health among keyworkers.

First, our data were from a non-probability sample. Despite the effort to make our sample representative of the working population in England by weighting, there is still the possibility of potential biases because of omitting other demographic factors that could be associated with survey participation in the weighting process. Second, we were only able to analyse data with respondents who reported themselves in government identified ‘keyworker’ roles at the start of the pandemic, but the definition of this changed throughout the pandemic. Future study is required to capture how changes in keyworker status designation may have had an impact on the mental health of these groups. Moreover, we lacked data on participants’ mental health prior to the COVID-19 pandemic. It therefore remains unclear whether the levels of depressive and anxiety symptoms had already been consistently high among the keyworkers in sectors such as utility, food chain, transport and delivery prior to COVID-19, or whether the conditions exacerbated their mental health during the pandemic.

Future research is encouraged to look at the longer-term mental health trajectories of keyworker in these groups, including after the current pandemic is under control. Although because of our large sample size we were able to examine more keyworker categories than prior studies, there was still some heterogeneity within the keyworker groups in our study. As a result of data limitations, we also were unable to include information on the front-line nature of keyworkers’ roles, which will be an important factor to explore in future studies. Finally, we were unable to account for whether and to what extent individual keyworkers within each category interfaced directly with the public, which might affect their levels of depression and anxiety.^5,6,17^

### Implications

Our findings indicate that the mental health of individuals in keyworker roles, such as essential services, that have been less visible than health and social care has been worst affected during the COVID-19 pandemic, suggesting the potential importance of mental health screening in at-risk occupations during pandemics. More mental health support will therefore be needed to deal with these symptoms as the pandemic eases. There is also a need for more fine-grained analyses of the mental health of different types of keyworkers, particularly those working in utility, transport and food chain roles to identify individuals in specific occupations who will need this support most. Future research should also seek to understand ways in which workplace measures to mitigate risk may have been inadequate during the current pandemic, to inform policies for future pandemics.

## Supplementary Material

Supplementary Material

## Figures and Tables

**Table 1:** Descriptive statistics of the total analytical sample and by keyworker status (weighted)

	Total, %		Non-keyworker, %	Health/social care, %	Teacher/chilh care, %	Public service, %	Essential services keyworkers, ^a^ %
	(n = 21 874)		(n = 14 252)	(n = 3326)	(n = 1241)	(n = 1815)	(n = 1240)
	Raw	Weighteh	Weighteh	Weighteh	Weighteh	Weighteh	Weighteh
Gender							
Women	78.8	51.0	49.1	69.8	74.2	51.3	33.2
Men	21.2	49.0	50.9	30.2	25.8	48.7	66.8
Ethnicity							
Ethnic minority groups	5.4	10.9	10.7	15.6	16.5	10.6	5.1
White	94.6	89.1	89.3	84.4	83.5	89.4	94.9
Age, years							
18-29	6.7	15.3	15.9	19.0	16.2	11.9	9.8
30-45	35.0	36.9	36.5	36.9	40.0	35.2	39.6
46-59	42.7	34.4	33.1	31.7	37.7	42.0	38.2
≥60	15.7	13.4	14.6	12.4	6.2	10.9	12.4
Education							
Low (up to GCSE)^b^	9.6	28.9	27.4	24.6	16.8	30.6	46.0
Medium (A-levels or equivalent)^b^	15.2	31.7	30.8	28.3	31.6	33.5	39.0
High (university degree or above)	75.2	39.4	41.9	47.1	51.6	35.8	15.0
Physical health diagnosis							
Yes	31.7	31.4	30.5	35.5	24.0	34.1	32.9
No	68.3	68.6	69.5	64.5	76.0	65.9	67.1
Mental health diagnosis							
Yes	16.4	16.7	16.0	21.0	15.8	16.7	16.1
No	83.6	83.3	84.0	79.0	84.2	83.3	83.9

a Essential services keyworkers includes utility worker (for example energy, sewerage, postal service), public safety or national security workers, workers involved in medicines or protective equipment production or distribution, transport workers still travelling in to work, and food chain workers (for example production, sale, delivery).

b General Certificate of Secondary Education (GCSE) typically taken at the age of 15 or 16; Advanced Level qualifications (A-levels) and other equivalent educational qualifications that are not part of higher education (typically age 16–19).

## Data Availability

Anonymous data will be made available in early 2022.
