# Supplementary material for: Comparing the mental health trajectories of four different types of keyworkers with non-keyworkers: 12-month follow-up observational study of 21 874 adults in England during the COVID-19 pandemic
Source: Br J Psychiatry. Author manuscript; Available in PMC 2022 Jul 20. (PMC7613104; doi:10.1192/bjp.2021.205)
Supplement: Supplementary Material [file EMS150148-supplement-Supplementary_Material.zip › S0007125021002051sup001.pdf]

Supplementary Materials

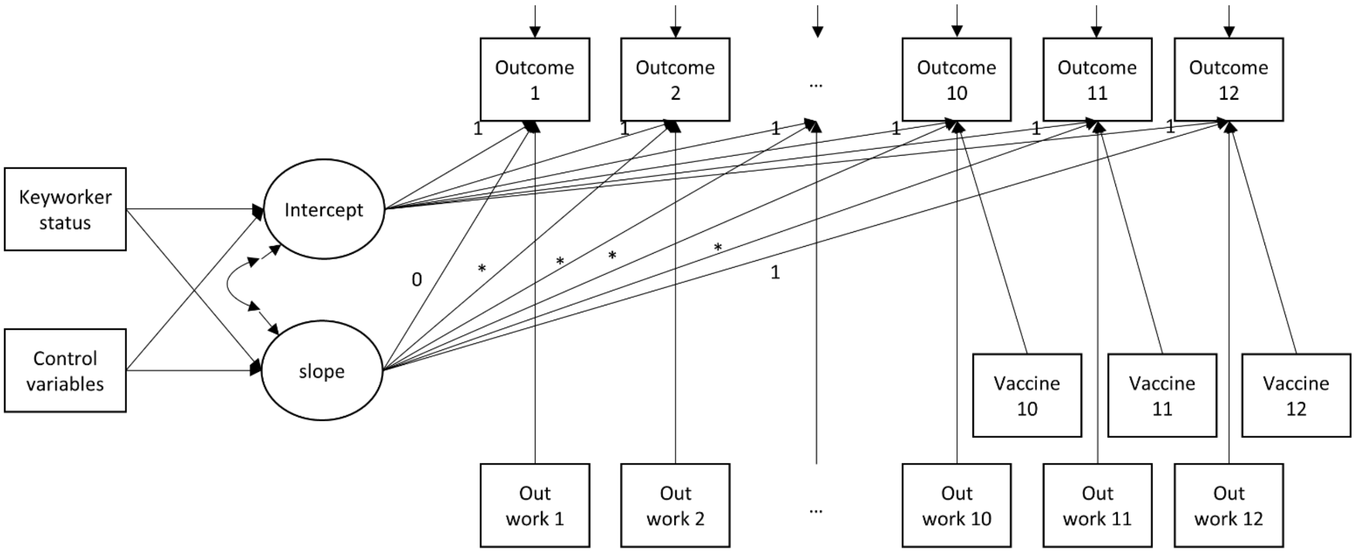

Figure S1. Model specification for the full model including both time-invariant and time-varying covariates

Notes: ‘Out work’ represents the time-varying variable indicating if participants went to work outside. ‘Vaccine’ is another time-varying variable which was available only from week 10 onwards.

Table S1. Dates corresponding to different time points (months)

Date	Month
21/03/20-17/04/20	1
18/04/20-15/05/20	2
16/05/20-12/06/20	3
13/06/20-10/07/20	4
11/07/20-07/08/20	5
24/08/20-20/09/20	6
21/09/20-18/10/20	7
19/10/20-15/11/20	8
16/11/20-13/12/20	9
14/12/20-10/01/21	10
11/01/21-07/02/21	11
08/02/21-22/02/21	12

Table S2. Percentages of participants with at least one wave of follow-up in each month

Month	Total	Non-key	Health/ social care	Teacher/ childcare	Public service	Essential services key workers
1	100.0%	100.0%	100.0%	100.0%	100.0%	100.0%
2	100.0%	100.0%	100.0%	100.0%	100.0%	100.0%
3	93.2%	93.3%	92.4%	92.7%	94.0%	92.4%
4	93.8%	94.0%	94.1%	93.7%	92.7%	92.6%
5	90.2%	90.6%	89.1%	87.9%	90.3%	90.4%
6	87.5%	87.9%	86.4%	85.4%	87.9%	86.9%
7	90.6%	90.9%	89.2%	91.4%	90.4%	89.6%
8	91.3%	91.3%	91.2%	91.7%	91.5%	90.9%
9	93.5%	93.6%	93.6%	93.3%	93.7%	92.3%
10	95.4%	95.6%	95.1%	95.2%	95.4%	93.3%
11[†]	44.3%	44.2%	44.3%	44.8%	43.4%	46.8%

Notes: [†] The low percentage of follow up for month 11 is due to incompletion of month 12 data collection

Table S3. Descriptive statistics by key worker status (unweighted)

	Non-key worker N=14,252	Health/ social care N=3,326	Teacher/ childcare N=1,241	Public service N=1,815	Essential services key workers[a] N=1,240
**Gender**					
Women	77.0%	88.9%	89.8%	78.6%	62.3%
Men	23.0%	11.1%	10.2%	21.4%	37.7%
**Ethnicity**					
Ethnic minority groups	5.5%	6.1%	5.9%	5.1%	2.8%
White	94.5%	93.9%	94.1%	94.9%	97.2%
**Age**					
18-29	6.8%	7.5%	5.9%	5.6%	5.6%
30-45	35.3%	34.6%	36.5%	32.5%	35.0%
46-59	40.9%	43.7%	50.9%	47.8%	44.5%
60+	17.0%	14.2%	6.7%	14.2%	14.9%
**Education**					
Low (Up to GCSE)[b]	8.8%	8.4%	5.9%	10.4%	24.4%
Medium (A-levels or equivalent)[b]	14.4%	12.6%	14.8%	17.4%	28.3%
High (Degree or above)	76.8%	79.0%	79.3%	72.2%	47.3%
**Physical health diagnosis**					
Yes	31.0%	36.2%	24.3%	33.4%	33.1%
No	69.0%	63.8%	75.7%	66.6%	66.9%
**Mental health diagnosis**					
Yes	15.7%	18.9%	16.3%	17.7%	15.0%
No	84.3%	81.1%	83.7%	82.3%	85.0%

Notes: [a] Essential services key workers includes utility worker (e.g. energy, sewerage, postal service), public safety or national security workers, workers involved in medicines or protective equipment production or distribution, transport workers still travelling in to work, and food chain workers (e.g. production, sale, delivery). [b] GCSE: General Certificate of Secondary Education typically taken at the age of 15 or 16; A-levels: Advanced Level qualifications, post-compulsory educational qualifications that are not part of higher education (typically age 16-19)

Table S4 Descriptive statistics of categorised depressive and anxiety symptoms at baseline and last wave (weighted)

	Total N=21,874	Non-key worker N=14,252	Health/ social care N=3,326	Teacher/ childcare N=1,241	Public service N=1,815	Essential services key workers N=1,240
**Depression (month 1)**						
Minimal depression (0-4)	47.9%	49.3%	41.1%	43.7%	48.0%	48.2%
Mild depression (5-9)	30.7%	29.9%	33.7%	39.3%	31.9%	28.2%
Moderate depression (10-14)	14.2%	14.4%	14.8%	12.0%	12.9%	14.4%
Moderately severe depression (15-19)	5.4%	4.8%	8.1%	2.6%	5.1%	7.3%
Severe depression (20-27)	1.8%	1.6%	2.3%	2.3%	2.1%	1.9%
**Depression (month 12)**						
Minimal depression (0-4)	52.6%	53.0%	48.9%	59.7%	51.1%	53.1%
Mild depression (5-9)	29.1%	29.8%	27.8%	32.1%	31.6%	23.1%
Moderate depression (10-14)	10.2%	10.2%	9.3%	7.1%	11.8%	10.9%
Moderately severe depression (15-19)	5.4%	4.7%	7.9%	1.0%	3.7%	10.0%
Severe depression (20-27)	2.8%	2.4%	6.1%	0.2%	1.8%	2.9%
**Anxiety (month 1)**						
Minimal anxiety (0-4)	57.8%	59.1%	52.2%	54.7%	57.3%	57.6%
Mild anxiety (5-9)	25.1%	24.5%	28.0%	27.3%	24.8%	25.4%
Moderate anxiety (10-14)	11.2%	11.1%	11.7%	12.9%	11.1%	10.6%
Severe anxiety (15-21)	5.9%	5.3%	8.1%	5.1%	6.7%	6.5%
**Anxiety (month 12)**						
Minimal anxiety (0-4)	67.1%	67.1%	63.6%	78.4%	69.1%	65.1%
Mild anxiety (5-9)	21.6%	22.0%	22.8%	15.9%	23.1%	19.1%
Moderate anxiety (10-14)	7.3%	7.0%	8.8%	4.1%	4.9%	10.1%
Severe anxiety (15-21)	4.0%	3.9%	4.8%	1.6%	2.8%	5.7%

Table S5 Results from the latent growth models on depressive and anxiety symptoms (unstandardised estimates)

	Depressive symptoms						Anxiety symptoms					
	Model I			Model II			Model I			Model II		
	Coef.	SE	p	Coef.	SE	p	Coef.	SE	p	Coef.	SE	p
**Main structure**												
Intercept	6.38	0.27	<0.001	5.79	0.86	<0.001	4.81	0.25	<0.001	4.03	0.62	<0.001
Slope	-0.95	0.18	<0.001	0.00	0.80	0.996	-0.80	0.21	<0.001	0.23	0.74	0.760
**Slope structure**												
Month 1	0.00	--	--	0.00	--	--	0.00	--	--	0.00	--	--
Month 2	-0.13	0.04	0.003	-0.26	0.11	0.020	0.06	0.03	0.032	-0.06	0.09	0.547
Month 3	0.35	0.05	<0.001	-0.33	0.20	0.090	0.43	0.04	<0.001	-0.09	0.18	0.640
Month 4	0.70	0.06	<0.001	0.02	0.22	0.925	0.69	0.05	<0.001	0.16	0.23	0.498
Month 5	1.03	0.08	<0.001	0.41	0.14	0.004	0.88	0.06	<0.001	0.34	0.24	0.166
Month 6	1.17	0.09	<0.001	0.65	0.17	<0.001	0.99	0.06	<0.001	0.63	0.23	0.007
Month 7	1.24	0.08	<0.001	0.90	0.18	<0.001	1.04	0.05	<0.001	0.77	0.23	0.001
Month 8	1.22	0.07	<0.001	1.20	0.17	<0.001	1.05	0.05	<0.001	1.03	0.18	<0.001
Month 9	1.14	0.06	<0.001	1.12	0.13	<0.001	1.07	0.04	<0.001	1.16	0.12	<0.001
Month 10	1.10	0.06	<0.001	1.19	0.12	<0.001	0.97	0.04	<0.001	1.17	0.09	<0.001
Month 11	1.00	0.05	<0.001	1.07	0.11	<0.001	0.99	0.03	<0.001	1.05	0.10	<0.001
Month 12	1.00	--	--	1.00	--	--	1.00	--	--	1.00	--	--
**Predictors of the intercept**												
Health/social care (vs. non-key worker)	*0.23*	*0.17*	*0.166*	*0.03*	*0.55*	*0.958*	*0.05*	*0.15*	*0.733*	*0.06*	*0.41*	*0.883*
Teacher/childcare (vs. non-key worker)	*-0.30*	*0.21*	*0.157*	*-0.83*	*0.41*	*0.042*	*0.08*	*0.22*	*0.732*	*-0.29*	*0.29*	*0.330*
Public service (vs. non-key worker)	*0.16*	*0.22*	*0.465*	*-0.63*	*0.50*	*0.212*	*0.15*	*0.20*	*0.474*	*-0.50*	*0.41*	*0.232*
Essential services (vs. non-key worker)	*0.09*	*0.23*	*0.685*	*1.98*	*0.58*	*0.001*	*0.25*	*0.21*	*0.230*	*1.17*	*0.46*	*0.011*
Women (vs. men)	0.91	0.12	<0.001	0.44	0.26	0.088	1.03	0.11	<0.001	0.66	0.23	0.005
Ethnic minority groups (vs. white)	0.37	0.21	0.078	-0.07	0.73	0.921	0.28	0.22	0.196	-0.73	0.56	0.196
Age 30-45 (vs. 18-29)	-0.89	0.20	<0.001	-0.68	0.74	0.358	-0.67	0.19	0.001	-0.28	0.54	0.602
Age 46-59 (vs. 18-29)	-2.01	0.21	<0.001	-2.14	0.75	0.004	-1.78	0.20	<0.001	-1.37	0.53	0.010
Age 60+ (vs. 18-29)	-3.03	0.24	<0.001	-3.14	0.77	<0.001	-2.75	0.22	<0.001	-2.36	0.55	<0.001
Education medium (vs. low)	-0.27	0.18	0.129	-0.36	0.39	0.356	-0.35	0.16	0.029	-0.55	0.33	0.092
Education high (vs. low)	-0.92	0.17	<0.001	-0.78	0.41	0.057	-0.59	0.15	<0.001	-0.52	0.33	0.119
Physical health diagnosis (vs. none)	1.16	0.13	<0.001	0.82	0.31	0.007	0.80	0.12	<0.001	0.56	0.25	0.024
Mental health diagnosis (vs. none)	5.16	0.19	<0.001	4.87	0.64	<0.001	4.41	0.17	<0.001	3.65	0.50	<0.001
**Predictors of the slope**												
Health/social care (vs. non-key worker)	*0.16*	*0.12*	*0.191*	*-0.29*	*0.28*	*0.290*	*-0.01*	*0.12*	*0.918*	*-0.32*	*0.27*	*0.230*

Teacher/childcare (vs. non-key worker)	*-0.29*	*0.17*	*0.088*	*-0.26*	*0.29*	*0.362*	*-0.29*	*0.18*	*0.108*	*-0.48*	*0.26*	*0.059*
Public service (vs. non-key worker)	*0.03*	*0.12*	*0.829*	*-0.37*	*0.21*	*0.079*	*-0.12*	*0.13*	*0.368*	*-0.38*	*0.20*	*0.063*
Essential services (vs. non-key worker)	*0.35*	*0.13*	*0.006*	*-0.66*	*0.39*	*0.091*	*0.14*	*0.15*	*0.363*	*-0.19*	*0.35*	*0.580*
Women (vs. men)	-0.33	0.08	<0.001	-0.21	0.17	0.211	-0.30	0.09	0.001	-0.11	0.17	0.520
Ethnic minority (vs. white)	0.08	0.13	0.547	-0.22	0.73	0.757	0.08	0.15	0.593	0.20	0.53	0.707
Age 30-45 (vs. 18-29)	0.02	0.14	0.894	-0.37	0.64	0.559	-0.14	0.16	0.392	-0.36	0.58	0.539
Age 46-59 (vs. 18-29)	0.27	0.14	0.053	-0.03	0.65	0.969	0.20	0.16	0.216	-0.17	0.61	0.778
Age 60+ (vs. 18-29)	0.45	0.15	0.004	-0.01	0.68	0.985	0.40	0.18	0.025	-0.22	0.63	0.724
Education medium (vs. low)	0.26	0.11	0.014	0.12	0.25	0.645	0.35	0.12	0.005	0.16	0.22	0.464
Education high (vs. low)	0.27	0.10	0.005	0.33	0.26	0.206	0.30	0.12	0.010	0.21	0.24	0.367
Physical health diagnosis (vs. none)	0.03	0.08	0.737	0.04	0.20	0.850	0.02	0.09	0.841	-0.04	0.17	0.822
Mental health diagnosis (vs. none)	-0.13	0.12	0.254	-0.08	0.30	0.787	-0.21	0.14	0.117	0.12	0.29	0.674
**Depressive/anxiety symptoms ON Out for work**												
Month 1				0.48	0.18	0.008				0.79	0.20	<0.001
Month 2				0.09	0.19	0.639				0.29	0.18	0.111
Month 3				-0.18	0.17	0.302				-0.07	0.16	0.672
Month 4				-0.45	0.12	<0.001				-0.34	0.10	0.001
Month 5				-0.96	0.12	<0.001				-0.65	0.08	<0.001
Month 6				-1.19	0.18	<0.001				-0.70	0.13	<0.001
Month 7				-0.76	0.17	<0.001				-0.30	0.15	0.052
Month 8				0.12	0.18	0.509				-0.04	0.18	0.819
Month 9				0.06	0.16	0.704				0.12	0.19	0.534
Month 10				0.08	0.17	0.646				0.09	0.19	0.633
Month 11				0.05	0.18	0.788				0.13	0.19	0.508
Month 12				-0.10	0.18	0.557				-0.16	0.16	0.306
**Depressive/anxiety symptoms ON vaccine status**												
Month 10				0.49	0.65	0.451				2.56	2.43	0.290
Month 11				-0.13	0.21	0.559				-0.07	0.17	0.700
Month 12				0.29	0.17	0.088				0.08	0.17	0.630
**Covariance structure**												
Intercept WITH slope	-2.37	0.25	<0.001	-1.34	0.48	0.005	-3.46	0.29	<0.001	-0.77	0.73	0.291
**Model fit indices**												
RMSEA		0.03			0.04			0.03			0.03	
CFI		0.93			0.91			0.94			0.91	
SRMR		0.03			0.03			0.02			0.03	

Notes: RMSEA stands for Root Mean Square Error of Approximation, CFI for Comparative Fit Index, and SRMR for Standardized Root Mean Square Residual

Table S6 Results from the latent growth models on depressive and anxiety symptoms (standardised estimates)

	Depressive symptoms						Anxiety symptoms					
	Model I			Model II			Model I			Model II		
	β	SE	p	β	SE	p	β	SE	p	β	SE	p
**Main structure**												
Intercept	1.26	0.05	1.26	1.31	0.17	<0.001	1.04	0.05	<0.001	1.10	0.16	<0.001
Slope	-0.42	0.08	-0.42	0.00	0.44	0.996	-0.30	0.08	<0.001	0.13	0.42	0.762
**Slope structure**												
Month 1	0.00	--	--	0.00	0.00	--	0.00	--	--	0.00	--	--
Month 2	-0.05	0.02	0.001	-0.10	0.04	0.012	0.03	0.02	0.037	-0.03	0.04	0.522
Month 3	0.15	0.02	<0.001	-0.13	0.07	0.069	0.24	0.02	<0.001	-0.04	0.08	0.624
Month 4	0.30	0.02	<0.001	0.01	0.08	0.926	0.39	0.02	<0.001	0.07	0.11	0.522
Month 5	0.43	0.02	<0.001	0.16	0.06	0.008	0.49	0.02	<0.001	0.15	0.12	0.213
Month 6	0.46	0.02	<0.001	0.24	0.07	<0.001	0.51	0.02	<0.001	0.27	0.12	0.026
Month 7	0.49	0.02	<0.001	0.33	0.07	<0.001	0.54	0.02	<0.001	0.32	0.12	0.006
Month 8	0.48	0.02	<0.001	0.44	0.06	<0.001	0.54	0.02	<0.001	0.42	0.09	<0.001
Month 9	0.45	0.02	<0.001	0.41	0.05	<0.001	0.55	0.02	<0.001	0.47	0.07	<0.001
Month 10	0.44	0.02	<0.001	0.43	0.04	<0.001	0.50	0.02	<0.001	0.47	0.05	<0.001
Month 11	0.39	0.02	<0.001	0.38	0.04	<0.001	0.51	0.02	<0.001	0.42	0.05	<0.001
Month 12	0.39	0.03	<0.001	0.36	0.04	<0.001	0.52	0.03	<0.001	0.40	0.06	<0.001
**Predictors of the intercept**												
Health/social care (vs. non-key worker)	0.05	0.03	0.166	0.01	0.12	0.958	0.01	0.03	0.733	0.02	0.11	0.883
Teacher/childcare (vs. non-key worker)	-0.06	0.04	0.157	-0.19	0.09	0.042	0.02	0.05	0.732	-0.08	0.08	0.330
Public service (vs. non-key worker)	0.03	0.04	0.464	-0.14	0.11	0.211	0.03	0.04	0.474	-0.14	0.11	0.232
Essential services (vs. non-key worker)	0.02	0.05	0.686	0.45	0.13	0.001	0.06	0.05	0.230	0.32	0.12	0.010
Women (vs. men)	0.18	0.02	<0.001	0.10	0.06	0.089	0.22	0.03	<0.001	0.18	0.06	0.005
Ethnic minority groups (vs. white)	0.07	0.04	0.077	-0.02	0.17	0.921	0.06	0.05	0.195	-0.20	0.15	0.196
Age 30-45 (vs. 18-29)	-0.18	0.04	<0.001	-0.16	0.17	0.350	-0.14	0.04	<0.001	-0.08	0.15	0.600
Age 46-59 (vs. 18-29)	-0.40	0.04	<0.001	-0.48	0.16	0.002	-0.39	0.04	<0.001	-0.37	0.14	0.008
Age 60+ (vs. 18-29)	-0.60	0.05	<0.001	-0.71	0.16	<0.001	-0.60	0.05	<0.001	-0.64	0.14	<0.001
Education medium (vs. low)	-0.05	0.04	0.128	-0.08	0.09	0.352	-0.08	0.04	0.029	-0.15	0.09	0.087
Education high (vs. low)	-0.18	0.03	<0.001	-0.18	0.09	0.048	-0.13	0.03	<0.001	-0.14	0.09	0.110
Physical health diagnosis (vs. none)	0.23	0.03	<0.001	0.19	0.07	0.008	0.17	0.03	<0.001	0.15	0.07	0.025
Mental health diagnosis (vs. none)	1.02	0.03	<0.001	1.10	0.12	<0.001	0.95	0.03	<0.001	0.99	0.12	<0.001
**Predictors of the slope**												
Health/social care (vs. non-key worker)	0.07	0.05	0.187	-0.16	0.15	0.279	-0.01	0.05	0.918	-0.18	0.14	0.205

Teacher/childcare (vs. non-key worker)	-0.13	0.08	0.091	-0.15	0.15	0.343	-0.11	0.07	0.108	-0.27	0.12	0.028
Public service (vs. non-key worker)	0.01	0.05	0.829	-0.20	0.11	0.064	-0.05	0.05	0.368	-0.21	0.11	0.050
Essential services (vs. non-key worker)	0.15	0.06	0.006	-0.36	0.21	0.076	0.05	0.06	0.365	-0.11	0.19	0.569
Women (vs. men)	-0.15	0.03	<0.001	-0.12	0.09	0.206	-0.11	0.03	0.001	-0.06	0.09	0.507
Ethnic minority (vs. white)	0.04	0.06	0.546	-0.12	0.40	0.760	0.03	0.06	0.594	0.11	0.29	0.700
Age 30-45 (vs. 18-29)	0.01	0.06	0.894	-0.21	0.35	0.558	-0.05	0.06	0.390	-0.20	0.33	0.542
Age 46-59 (vs. 18-29)	0.12	0.06	0.057	-0.01	0.36	0.969	0.08	0.06	0.217	-0.10	0.34	0.779
Age 60+ (vs. 18-29)	0.20	0.07	0.004	-0.01	0.37	0.985	0.15	0.07	0.025	-0.12	0.35	0.727
Education medium (vs. low)	0.12	0.05	0.012	0.06	0.14	0.644	0.13	0.05	0.004	0.09	0.12	0.442
Education high (vs. low)	0.12	0.04	0.005	0.18	0.14	0.200	0.11	0.04	0.009	0.12	0.12	0.337
Physical health diagnosis (vs. none)	0.01	0.03	0.737	0.02	0.11	0.851	0.01	0.03	0.841	-0.02	0.10	0.820
Mental health diagnosis (vs. none)	-0.06	0.05	0.252	-0.04	0.16	0.787	-0.08	0.05	0.111	0.07	0.16	0.677
**Depressive/anxiety symptoms ON Out for work**												
Month 1				0.09	0.04	0.008				0.18	0.05	<0.001
Month 2				0.02	0.04	0.640				0.07	0.05	0.109
Month 3				-0.04	0.04	0.303				-0.02	0.04	0.672
Month 4				-0.10	0.03	<0.001				-0.09	0.03	0.001
Month 5				-0.20	0.03	<0.001				-0.16	0.02	<0.001
Month 6				-0.24	0.04	<0.001				-0.17	0.03	<0.001
Month 7				-0.15	0.04	<0.001				-0.07	0.04	0.054
Month 8				0.02	0.04	0.507				-0.01	0.04	0.820
Month 9				0.01	0.03	0.704				0.03	0.04	0.534
Month 10				0.02	0.03	0.646				0.02	0.04	0.633
Month 11				0.01	0.04	0.788				0.03	0.04	0.509
Month 12				-0.02	0.04	0.557				-0.04	0.04	0.305
**Depressive/anxiety symptoms ON vaccine status**												
Month 10				0.10	0.13	0.451				0.57	0.54	0.290
Month 11				-0.02	0.04	0.558				-0.02	0.04	0.700
Month 12				0.06	0.03	0.086				0.02	0.04	0.630
**Covariance structure**												
Intercept WITH slope	-0.24	0.02	<0.001	-0.19	0.06	0.001	-0.32	0.02	<0.001	-0.13	0.11	0.229
**Model fit indices**												
RMSEA		0.03			0.04			0.03			0.03	
CFI		0.93			0.91			0.94			0.91	
SRMR		0.03			0.03			0.02			0.03	

Notes: RMSEA stands for Root Mean Square Error of Approximation, CFI for Comparative Fit Index, and SRMR for Standardized Root Mean Square Residual
